# Modeling of hexamethylene diisocyanate and psychrometric parameters and other effective factors in the polyurethane factories

**DOI:** 10.4103/0019-5278.75693

**Published:** 2010

**Authors:** Mirtaghi Mirmohammadi, M. H. Ibrahim, J. N. Saraji

**Affiliations:** Department of Occupational Health, Mazandaran University of Medical Sciences, Sari, Iran

**Keywords:** Diisocyanate, dry bulb temperature, hexamethylene diisocyanate concentration, regression, relative humidity

## Abstract

**Background:**

Diisocyanates are widely used in surface coatings, polyurethane (PUR) foams, adhesives, resins, elastomers, binders, and sealants. Isocyanate exposure is irritative to the skin, mucous membranes, eyes, and respiratory tract. The most common adverse health outcome associated with isocyanate exposure is asthma due to sensitization.

**Objective:**

The goal of this study is to find statistical predictive model to determine the relationship between airborne hexamethylene diisocyanate (HDI) and selective psychrometric variables.

**Materials and Methods:**

All air samplers (by midget impinger) were collected by mini personal sampler pump fixed to work stations near the source of pollution. The air samples and psychrometric parameters were separately collected and determined in a working shift for three periods of 2 h, each at a flow rate of 2 l/min in an impinger containing a solution of reagent of dimethyl sulfoxide in tryptamine [US National Instituteof Occupational Safety and Health (NIOSH), 1994].

**Results:**

There was a significant correlation between HDI concentration and relative humidity and dry bulb temperature (*P* < 0.05). No significant correlation was seen between altitude and dimension of PUR factories (*P* > 0.05).

**Conclusions:**

The finding of the study may be a useful initial tool in estimating possible HDI pollution situation in the PUR workplaces, based on simple psychrometric factors (indoor air temperature and relative humidity).

## INTRODUCTION

Nowadays, the modern work style in the industries has brought into use all the chemical materials and high toxic materials in production process. Use of chemical materials in the workplace causes indoor air pollution problems. Aromatic diisocyanates such as methylene diphenyl diisocyanate (HDI) are extensively used in the manufacture of polyurethane (PUR) products.[1] The main PUR products are flexible and rigid foam; other applications are as adhesives, sealants, binders, surface coatings and more. The global production of diisocyanates is annually increasing. In Europe alone, it is estimated that over 70,000 employees are manufacturing isocyanates or PUR.[2] The numbers of workers further processing the PUR products are not known. The exposure to isocyanates is complex and different in different applications. The lower molecular weight isocyanates tend to volatilize at room temperature, creating a vapor inhalation hazard. Conversely, the higher molecular weight isocyanates do not readily volatilize at ambient temperatures, but are still an inhalation hazard if aerosolized or heated in the work environment. The latter is important since many reactions involving isocyanates are exothermic in nature, thus providing the heat for volatilization.[3] As exposure limits decrease, the volatility of solid materials becomes an issue. To reduce the vapor hazards associated with the lower molecular weight diisocyanates, prepolymer and polyisocyanate forms of these diisocyanates were developed and have replaced the monomers in many product formulations. An example is HDI, which consists of three molecules of HDI monomer joined together to form a higher molecular weight oligomer having similar characteristics to those found in the monomer.[4] The specific goal of this study is to answer the following question: How does the HDI concentration change with respect to psychrometric and factory factors?

The correlation and regression analysis techniques are useful in investigating these relationships. The relationship between meteorological parameters and pollutant concentrations has been well studied in different parts of the world for different time periods. A lot of research has been devoted to the study of the local climatic conditions in relation to the air quality. Dickson, for example, studied the relationship between precipitation and particulate matter in Nashville, whereas Sham used a linear correlation to model respirable dust particulate and average wind speed in Malaysia.

## MATERIALS AND METHODS

Of the PUR factories, 5 HDI factories were selected which had 450 workers, for this study; all of them manufactured foaming or PUR foams. The predominantly used isocyanate was HDI. There were 200 unexposed workers who worked in the office area. The sampling and analysis of isocyanates are divided into four steps: collection, derivatization, sample preparation and identification.[5] The samplers have been evaluated for aspiration efficiencies and internal losses. All of the samplers with midget impinger were connected to mini personal sampler pump fixed to workstations near the source of pollution. The air sample was collected for 2 h at a flow rate of 2 l/min, in the impinger containing a solution of reagent of dimethyl sulfoxide (DMSO) in tryptamine. Because DMSO is readily absorbed through the skin, US National Instituteof Occupational Safety and Health (NIOSH) recommends that DMSO impingers be used for area air sampling only. Reversed-phase high performance liquid chromatography (HPLC) has been the dominant separation technique used in isocyanate analysis.[6] The first step in the analysis of a solution is derivatization of isocyanates for the separation through HPLC, for their qualitative as well as quantitative analysis.[7] Air sampling was performed according to NIOSH method 5522 for isocyanate in air. Air samples were collectedat a flow rate of 2 l/min using a midget impinger sampler (SKC, Eighty Four, PA, USA) and personal sampling pumps were calibratedbefore and after sampling with a Dry Cal DC-Lite primary flow meter(Bios International Co., Pompton Plains, NJ, USA). A KNAUER HPLC was used for air analysis. The HPLC equipment consisted of a high-pressure pump, a variable-wavelength UV detector and a loop injector; HPLC columns were made of stainless steel (200 mm long × 5 mm in inside diameter) packed with 5 m Nucleosil C18. The air concentrations of HDI were monitored continuously by the midget impinger instrument using DMSO with tryptamine reagent and the sampling time for HPLC analyses was 1 h at an air flow rate of 2 l/min.

During air sampling for HDI concentration inside the factories, data for indoor relative humidity, indoor dry bulb temperature, altitude and dimension of factory were recorded. HDI was considered as a dependent variable and the rest were independent variables. These independent indoor air variables were divided into two groups. One was the indoor air psychrometric parameters involving the relative humidity (Rh, %) and the dry bulb temperature (Td, °C). The other group was the factory parameters involving the factory dimension in terms of factory space (D, m^3^) and the altitude of factory location (Alt, m).

Regression analysis procedure was used to state the statistical relationship between the variables and identify any meaningful relationship between those variables. Due to the fact that the number of independent was variables more than one, multiple linear regression analysis was used in the present analysis.[8] A multiple regression equation, which has four independent variables, was used, and it can be expressed as follows:

(1) $$Y\;=\;B_{0}\;+\;B_{1}X_{1}\;+\;B_{2}X_{2}\;+\;B_{3}X_{3}\;+\;B_{4}X_{4}\;+\;e_{i}$$

where

Y is the dependent variable (HDI concentration),

X_1_, X_2_, X_3_ and X_4_ are independent variables (relative humidity, dry bulb temperature, factory dimension and altitude) as the predictors in this model,

B_0_, B_1_, B_2_, B_3_ and B_4_ are the model coefficients, and e_i_ is the residual error.

The values for the constant and the coefficients are determined using the least-squares method which minimizes the error as “e” in the above regression equation. The significance levels of the constant and coefficients are statistically tested using *t*-distribution. The *R*^2^ (coefficient of determination) determines the direction and significance level of relation between the variables in the mathematical model and shows how much the dependent variable is affected by the independent variables.

All statistical analyses were performed using SPSS software, version 16. In this study, the alpha level was 0.05 (*P* ≤ 0.05), which was similar to other indoor air pollution studies.[9–11]

## RESULTS

Table 1 shows the maximum, minimum and mean values of indoor air independent variables with respect to HDI concentration in the PUR factories for 100 air samples which were gathered from workplaces separately by static sampling procedure. The lowest minimum HDI concentration in all the factories was 61 *µ*g/m^3^ and also the highest of the maximum HDI concentration was 96 *µ*g/m^3^. These values can be considered as high, when compared to NIOSH exposure limit of 35 *µ*g/m^3^. The mean HDI concentration was 78.8 *µ*g/m^3^, the mean indoor relative humidity was 37.7%, and the mean dry bulb temperature was 28.3°C. The size of workplace for the five PUR factories ranged from 5000 to 9800 m^3^ and the altitude of factories was from as low as 22 m to as high as 1200 m.

**Table 1:** Values of indoor air variables in the HDI polyurethane factories

Variables	Factory codes					Average
	H_1_	H_2_	H_3_	H_4_	H_5_
HDI concentration (*µ*g/m^3^), max	96	92	90	90	88	91.2
Min	67	66	66	64	61	64.8
Mean	82.2	79.8	78.7	77	76.7	78.8
Relative humidity (%), max	52	52	45	45	40	46.8
Min	32	31	31	31	31	31.2
Mean	40.5	40	37	37	34	37.7
Dry bulb temperature (°C), max	33	32	32	32	30	31.8
Min	25	24	24	24	23	24
Mean	29.3	27.8	28.7	28.7	27.1	28.3
Dimension of factory (m^3^)	5000	6100	7400	9000	9800	7460
Altitude (m)	1200	1200	1100	890	22	882

Max: maximum, min: minimum, NIOSH guideline value: 35(*µ*g/m^3^)

It has been summarized and stated in Table 1 that several factories show variable concentration of diisocyanates, with respect to relative humidities of each factory. The highest mean value HDI concentration was 82.2 *µ*g/m^3^ which corresponds to the highest mean relative humidity of 40.5% inside factory H_1_. Factories H_1_ and H_2_ were located in Tehran province. Factory H_2_ had the second highest mean HDI concentration and also the second highest mean relative humidity (79.8 *µ*g/m^3^ and 40%, respectively). Factory H_5_ had the lowest mean value HDI concentration which was 76.7 *µ*g/m^3^. Factory H_5_ was situated in Mazandaran province and this factory had the lowest mean relative humidity of 34%.

Table 1 also summarizes that several factories have variable concentration of diisocyanates with respect to different dry bulb temperature. In factory H_1_,the highest mean HDI concentration was 82.2 *µ*g/m^3^ and this corresponds to the highest mean dry bulb temperature of 29.3°C. Similarly, in factory H_5_, the lowest mean HDI concentration was 76.7 *µ*g/m^3^ which corresponds to the lowest mean dry bulb temperature 27.1°C.

### Multiple linear regression analyses

Multiple regression analyses were performed to determine the psychrometric variables and other factors that explained the most variance in the overall data. The results of application of correlation coefficients and regression analysis are listed in Tables 2 and 3.

**Table 2:** Correlation result for HDI concentration and psychrometric parameters and factory parameters

		HDI (*µ*g/m^3^)	RH (%)	Td (°C)	D (m^3^)	Alt (m)
HDI (*µ*g/m^3^)	Pearson Correlation	1	0.739	0.88	-0.887	0.85
	Sig. (2-tailed)	.	0.0001	0.0001	0.0001	0.0001
	N	100	100	100	100	100
RH (%)	Pearson Correlation	0.739	1	0.639	-0.687	0.744
	Sig. (2-tailed)	0.0001	.	0.0001	0.0001	0.0001
	N	100	100	100	100	100
Td (°C)	Pearson Correlation	0.88	0.639	1	-0.963	0.719
	Sig. (2-tailed)	0.0001	0.0001	.	0.0001	0.001
	N	100	100	100	100	100
D (m^3^)	Pearson Correlation	-0.887	-0.687	-0.963	1	-0.812
	Sig. (2-tailed)	0.0001	0.0001	0.0001	.	0.0001
	N	100	100	100	100	100
Alt (m)	Pearson Correlation	0.85	0.744	0.719	-0.812	1
	Sig. (2-tailed)	0.0001	0.0001	0.0001	0.0001	.
	N	100	100	100	100	100

Correlation is significant at the 0.05 level (2-tailed).

**Table 3:** Regression model summary of HDI

Model	r	*R^2^*	Adjusted *r^2^*
	0.915	0.837	0.83

Predictors: (constant), altitude (m), dimension of factory (m^3^), relative humidity (%), dry bulb temperature (°C)

#### Relative humidity

From Table 2, it is seen that relative humidity also correlates positively with HDI concentration. A significant relationship was found between HDI concentration and relative humidity in the PUR factories. The relative humidity in the study area was more than 34% on the average. Therefore, higher relative humidity corresponds to a greater concentration level of HDI in the PUR factories.

#### Dry bulb temperature

A correlation between HDI concentration and dry bulb temperature yields a significant positive relationship. This implies that increased dry bulb temperature in the workplaces corresponds to high levels of HDI concentration.

#### Dimension of workplace

No correlation was seen between HDI concentration and dimension of workplaces. The correlation coefficient, however, is less and statistically not significant at 0.142 level, but negative.

#### Altitude

The correlation between HDI concentration and altitude did not show any relationship. No significant relationship was found between HDI concentration and altitude.

The results imply that all or some of parameters (altitude, dimension of factory, relative humidity and dry bulb temperature) can be significant predictors of HDI concentration in the PUR workplaces.

Table 4 presents an analysis of variance for HDI concentration in the PUR factories. From the table, it can be seen that F is 121.9 which is significant at *P* < 0.05. We can conclude that the regression model predicts the concentration level of HDI significantly well.

**Table 4:** Regression model for HDI polyurethane factories’ factors

Model	Mean square	*F*	*P* value
Regression	1155.895	121.934	0.0001
Residual	9.48		
Total	0		

Predictors: (constant), altitude (m), dimension of factory (m^3^), relative humidity (%), dry bulb temperature (°C), dependent Variable: HDI concentration (*µ*g/m^3^)

Since the results of regression model test in Table 4 illustrate that the independent variables are significant predictors of HDI concentration, we can employ equation (1) to stand for the different psychrometric and factory parameters in order to measure the predictive regression correlation between the parameters and HDI concentration.

Table 5 shows the results of regression analysis between HDI concentration and PUR indoor air parameters. Both indoor relative humidity and dry bulb temperature can be seen to be significant predictors of HDI pollution (*P* < 0.05). Both these parameters fall under the psychrometric parameters group. The other two parameters (factory dimension and altitude) are the factory parameters and both are not significant (*P* > 0.05). This means that the size of HDI PUR factories and geographical positions of the factories in terms of altitude are not significant where the HDI pollution is concerned and are therefore taken out from the regression model.

**Table 5:** Result of regression analysis between HDI concentration and polyurethane indoor air parameters

Model	Coefficients		*T*	*P* value
	B	SE		
(Constant)	43.267	17.837	2.426	0.017
Relative humidity (%)	0.367	0.071	5.182	0.0001
Dry bulb temperature (°C)	1.112	0.399	2.785	0.006
Dimension of factory (m^3^)	–0.001	0.001	–1.479	0.142
Altitude (m)	0.003	0.002	1.475	0.144

Dependent Variable: HDI concentration (*µ*g/m^3^)

The independent variables (relative humidity and dry bulb temperature) were reproduced for the model to find the regression coefficients for HDI pollution in the PUR factories. The coefficients with respect to the constant, relative humidity and the dry bulb temperature as well as the collinearity statistics are shown in Table 6.

**Table 6:** The collinearity statistical model coefficients

Model	Coefficients		t	*P* value	Collinearity statistics	
	B	SE			Tolerance	VIF
(Constant)	27.771	2.638	10.528	0.0001		
Relative humidity (%)	0.368	0.067	5.454	0.0001	0.592	1.689
Dry bulb temperature (°C)	1.492	0.119	12.588	0.0001	0.592	1.689

Dependent variable: HDI concentration (*µ*g/m^3^)

Collinearity diagnostics was used in this study to find multicollinearity between dependent (HDI concentration) and independent variables (relative humidity and dry bulb temperature). Multicollinearity here refers to linear intercorrelation among psychrometric variables. If psychrometric variables (relative humidity, dry bulb temperature) correlate highly, they are redundant in the same model. The best regression models are those in which the each of the predictor variables correlate highly with the dependent (outcome) variable but correlate at most only minimally with each other.[12]

The two factory predictor variables (dimension of factory and altitude) were not found to be significant in the model. They were eliminated for making a new regression model based on relative humidity and dry bulb temperature. By replacing Y, X_1_ and X_2_ with HDI, Rh (relative humidity) and Td (dry bulb temperature), respectively, and eliminating X_3_ (factory dimension) and X_4_ (altitude) and also substituting the relevant coefficients from Table 6, equation (1) can be written as follows:

(2) HDI = 27.77 + 0.368Rh + 1.492Td

Equation (2) implies that relative humidity and dry bulb temperature affect diisocyanates pollutant concentration in the workplaces. The background HDI concentration was about 27.77 *µ*g/m^3^, as indicated by the value of the constant in the regression equation.

**Figure 1:**
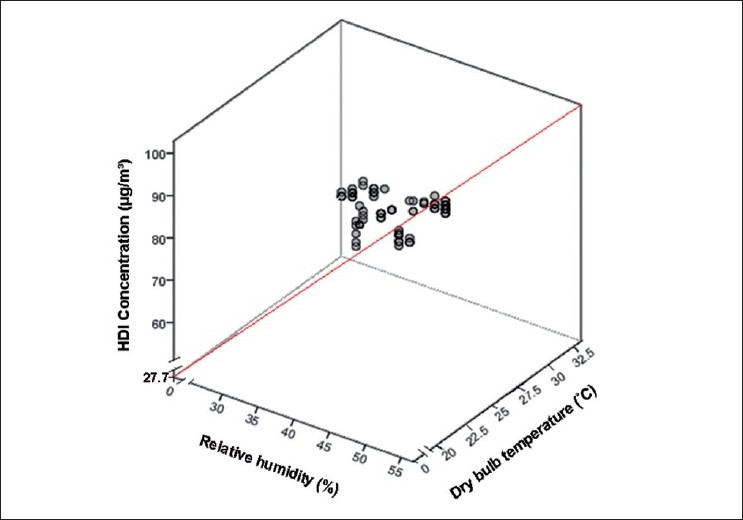
Relationship between HDI concentration and psychrometric parameters (relative humidity and dry bulb temperature)

Figure 1 shows the relationship between HDI concentration and psychrometric parameters (relative humidity and dry bulb temperature) in five PUR factories based on equation (2).

Both indoor air relative humidity and dry bulb temperature significantly contribute to the variability of the HDI concentration (*R*^2^ = 0.837) and both factors also show a straight positive relationship with the HDI concentration. This means that as the indoor relative humidity or the dry bulb temperature increases, the HDI concentration also increases. Equation (2) (also shown in Figure 1) suggests that the average background HDI concentration was about 27.77 *µ*g/m^3^, as indicated by the value of the constant in the regression equation.

## DISCUSSION

A positive relationship between temperature and HDI concentration can be expected as it was reported in the literature,[13] where an exponential increase of isocyanate emission rate was observed during curing of PUR adhesives. It was seen that in the range of typical room temperatures (20–30°C) there was only small effect on emission. However, at temperatures greater than 40°C, a clear increase in emission was noticed. The indoor temperatures within the factories ranged from 23 to 33°C. The linear relationship between temperature and HDI concentration is acceptable since the indoor temperatures were not greater than 40°C where exponential increase of isocyanate emission could occur. Vilhelm has found that dry bulb temperature affects diisocyanates pollutant generation speed and the volatility of the isocyanates emission into workplaces. They reported that the speed of the reaction also depends a great deal on temperature.[14]

As for the effects of relative humidity on isocyanate concentration, Ludwig and Urban observed that reactions of isocyanate groups with OH groups during cross-linking are inversely proportional to the relative humidity of the environment. They explained that the presence of competing reactions between water and isocyanate hinders the degree of cross-linking between them. Abram and Bowler studied the effect of relative humidity on the curing and dielectric properties of the PUR-based composites. They found that a PUR factory at 87% relative humidity gave more noticeable effect as compared to one at 37.7% RH. The electromagnetic properties of the polyurea/PUR-based composites studied were found to be strongly influenced by the presence of water vapor during the curing process, as evidenced by the significant difference in the real relative permittivity of samples cured in different RH environments.[15] This difference was caused primarily by water uptake into the polymer matrix. The RH alters the dielectric properties of the composite material due to its strong polar nature and high value of real relative permittivity. In this study, the RH was in the range of 31–52%. The working range for RH in this study is lower than that reported by Abram and Bowler for the noticeable effects (87% RH). Hence, the effect of RH is expected to be not as significant as to the effects of temperature.[16]

The relationship between psychrometric factors such as indoor temperature and relative humidity was extracted from some normal gas emission behavior in the workplaces and the relationship depended on the gas phase reaction of isocyanates.[17]

The mean isocyanate concentration in this study was 78.87 *µ*g/m^3^ (0.079 mg/m^3^). This is comparable to an investigation conducted in southern Australian auto body shops, which reported a geometric mean concentration of 0.07 mg/m^3^ isocyanates (range <0.01–3.5 mg/m^3^ NCO).[18] Swedish auto body shops’ exposures during spray paintingshowed higher values between 0.26 and 1.1 mg/m^3^.[19] A survey of Oregon auto body shops measureda geometric mean concentration of 0.35 mg/m^3^ isocyanate with a maximumof 4 mg/m^3^ isocyanate.[20] Maître *et al*. have reportedan arithmetic mean level of 0.33 mg/m^3^ isocyanate (range 0.05–0.65 mg/m^3^) in a French isocyanates spray factory. These findings suggest that the measurements in this research are comparable to other related literatures.[21] However, comparison of such results is quite difficult because the sampling time, sampling method and manufacturing process were totally different.[22]

Heitbrink *et al*. studied psychrometric factors and diisocyanates in different PUR factories. They stated that there is a relationship between high concentration of diisocyanates andexposures for workers.[23] In general, direct comparison of the results of present study with those of previous studies is quite difficult. Woskie *et al*. observed a number of reasons for this, such as often the sampling times are quite different, the results were described with a variety of descriptive statistics (arithmetic means, geometric means, etc.), the aims of the studies varied greatly (sampler comparisons, method evaluations, surveillance and epidemiology) and the studies were conducted in different countries, covering different periods.[24] Furthermore, in such studies, many different materials were used as calibration for standards and different units were used to express the exposure concentrations.

The psychrometric regression model obtained in this study was the same as that of dust correlation study performed in Malaysia[25] and almost close to that of PUR factories.[24]

The relationship between psychrometric factors such as indoor temperature, relative humidity and altitude was extracted from some normal gas emission behavior in the workplaces where it depended on the gas phase reaction of isocyanates.[16] The results from psychrometric factors and diisocyanates in this study indicated a strong dependence of emission rates on indoor temperature and diisocyanates and this result was the same as that of other studies conducted in different geographical places in Germany, in the current study, for at the result of this study showed diisocyanates had a negative relationship to altitude (based on gas law, altitude has a reverse relation with air pressure. This can be ascribed to the higher volatility of isocyanate pointed to this relationship as well.[8,26,27]

## CONCLUSIONS

The results of indoor air monitoring in all the five Iranian PUR factories showed high HDI concentration values, in comparison with NIOSH exposure limit of 35 *µ*g/m^3^. The HDI concentration ranged from 62.24 to 92.7 *µ*g/m^3^ with a mean HDI concentration of 78.8 *µ*g/m^3^.

Both indoor air relative humidity and temperature significantly contribute to the variability of the HDI concentration (*R*^2^ = 0.837) and both factors also showed a straight relationship with the HDI concentration. The size of factories and altitude at which the factories were located did not seem to be significant predictors of HDI concentration level in the factories. Multiple linear regressions were used to relate relative humidity and indoor air temperature to HDI, according to equation (2) as follows:

(2) HDI = 27.77 + 0.37Rh + 1.49Td

The background HDI concentration was about 27.77 *µ*g/m^3^, as indicated by the value of the constant in the regression equation.

The current work admittedly has limitations but may be a useful initial tool in estimating possible HDI pollution situation in the PUR workplaces, based on simple psychrometric factors (indoor air temperature and relative humidity). It can serve as a basis of future evaluation and intervention efforts to reduce the workers to isocyanate exposure.
